# Direct anterior approach for femoral osteotomy: a new surgical technique

**DOI:** 10.1093/jhps/hnaf041

**Published:** 2025-08-04

**Authors:** Frédéric Laude, Camille Vorimore

**Affiliations:** Clinique du Sport, 36 Boulevard Saint-Marcel, 75005 Paris, France; Clinique du Sport, 36 Boulevard Saint-Marcel, 75005 Paris, France

## Abstract

Femoral osteotomy is an effective surgical intervention for correcting torsional deformities that contribute to hip instability and femoroacetabular impingement. However, traditional osteotomy techniques require extensive soft tissue dissection, disrupt the femoral canal, and complicate future total hip arthroplasty. Additionally, traditional approaches frequently involve multiple incisions when combined with other procedures, prolonging recovery and increasing morbidity. The direct anterior approach (DAA) offers a minimally invasive alternative that preserves the femoral anatomy while enabling simultaneous intra-articular interventions. This technique provides access to the femoral neck for varisation, valgisation, and rotational osteotomies while preserving critical vascular structures. Moreover, the DAA facilitates concurrent hip arthroscopy, periacetabular osteotomy, and cartilage restoration procedures. We describe the DAA for femoral neck osteotomy to correct torsional deformities and discuss its potential benefits in optimizing surgical outcomes.

## INTRODUCTION

Excessive femoral anteversion can exacerbate hip instability due to inadequate femoral head coverage. Conversely, femoral retroversion can lead to anterior impingement, restricting functional flexion and limiting internal rotation [1, 2]. Both abnormalities are well-established risk factors for the development of hip osteoarthritis [3]. Femoral corrective osteotomy is an effective intervention for addressing these rotational deformities and can be performed at various levels, including intertrochanteric, subtrochanteric, diaphyseal, or supracondylar, using dedicated fixation systems [4–6]. However, conventional techniques significantly alter the shape of the femoral intramedullary canal, making future total hip replacement more complex [7]. Additionally, they provide limited access for concurrent intra-articular procedures and frequently require extensive soft tissue dissection. Thus, proximal femoral osteotomy is associated with a prolonged healing process, significant scarring, and a high likelihood of requiring implant removal [8, 9]. As a result, the use of femoral osteotomies has declined in current clinical practice [10].

To address these challenges, we propose a simplified, minimally invasive technique using the direct anterior approach (DAA).

## SURGICAL TECHNIQUE

### Pre-operative planning

In addition to anterior–posterior pelvic and false-profile radiographs, a low-dose pelvic CT scan, including both distal femoral condyles, is performed to assess femoral neck anteversion, with 3D reconstruction enabling precise identification of the cutting plane in relation to the intertrochanteric tubercle, which is palpable intraoperatively.

### Patient positioning

**Figure 1:**
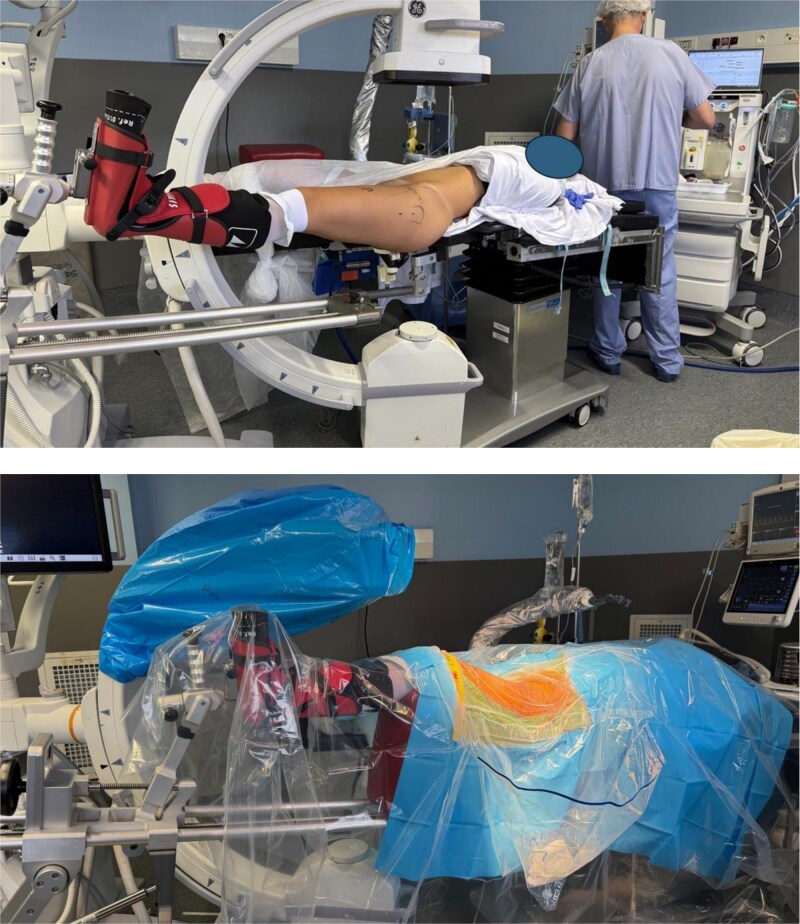
Patient installation for femoral osteotomy.

The patient is positioned on an orthopaedic traction table. The fluoroscopy unit is positioned between the patient’s legs (Fig. 1).

### Approach

**Figure 2:**
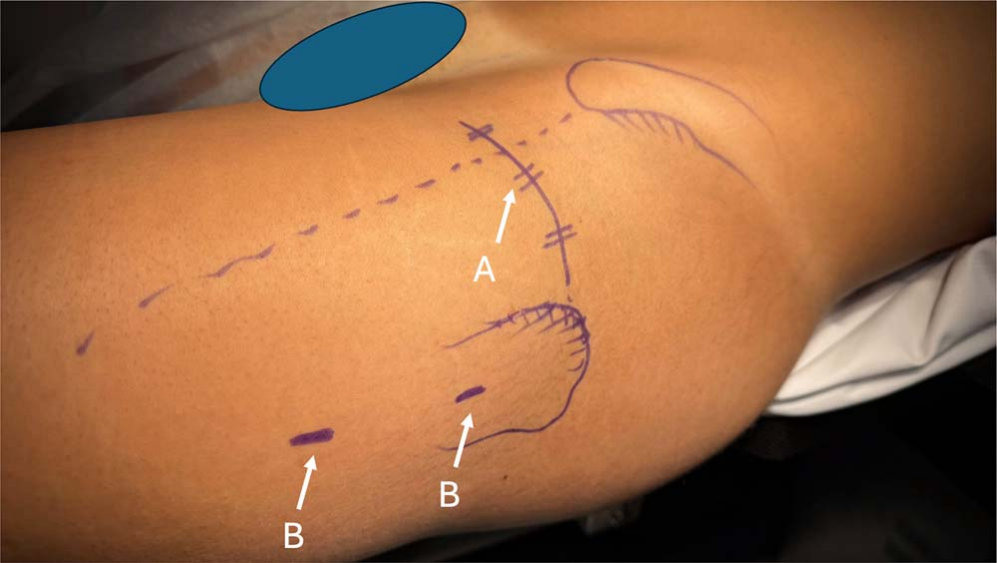
Anatomical landmark markings on skin: (A) DAA with bikini approach, (B) lateral fixation approach.

**Figure 3:**
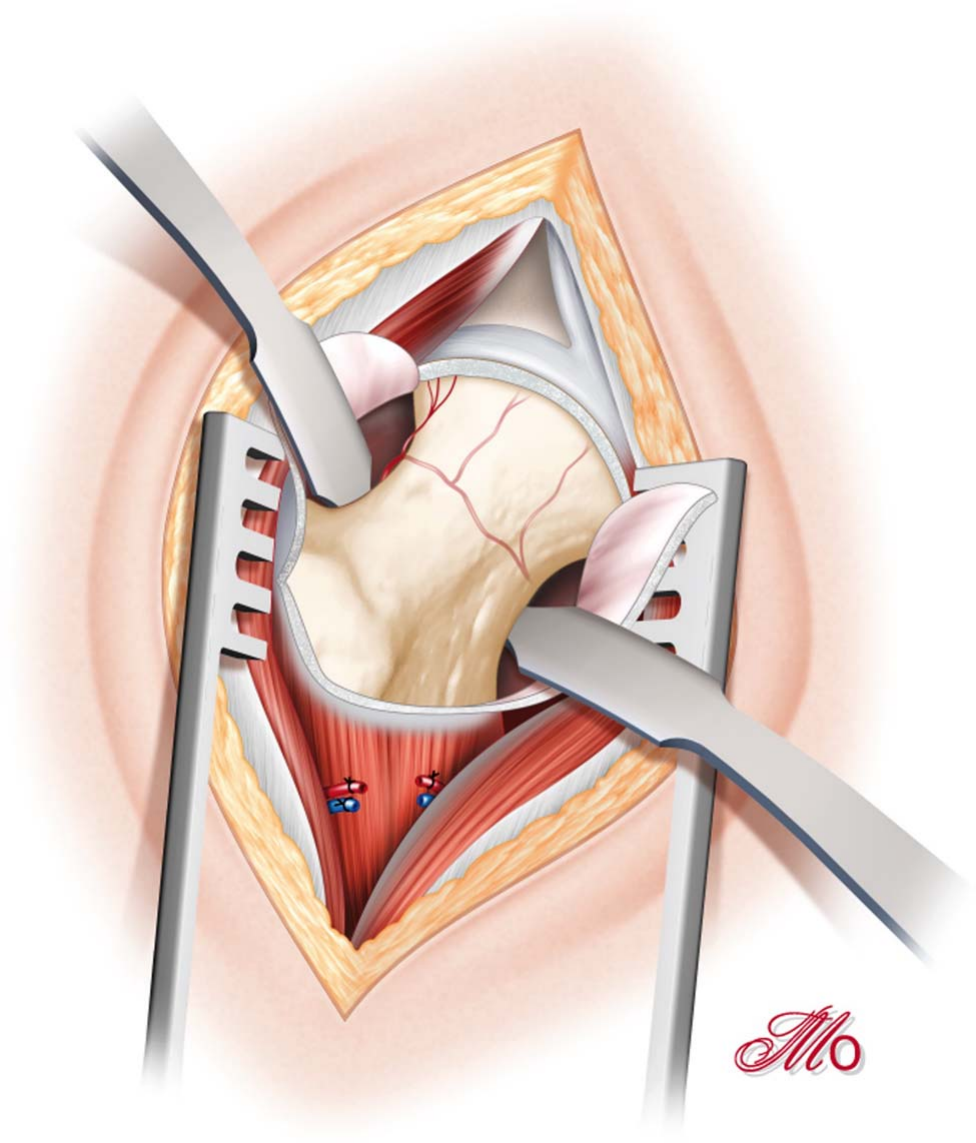
Femoral head exposure using the DAA.

The procedure begins with a DAA using a 5 cm minimally invasive incision between the tensor fasciae latae and the rectus femoris. A Bikini approach can also be used (Fig. 2). The innominate aponeurosis is opened, the anterior circumflex vascular bundle ligated, and the capsule incised in an inverted V-shape with a vertical cut parallel to the psoas and a horizontal cut parallel to the vastus lateralis. The vascular retinaculum supplying the femoral head can be observed slightly posteriorly on the upper surface of the femoral neck. If needed, the vastus lateralis can be partially released by a few millimetres to improve exposure (Fig. 3). At this stage, an arthroscope can be introduced into the joint either through the DDA or an additional portal. Arthroscopy is performed with air insufflation instead of irrigation.

### Femoral osteotomy

The DAA allows for both varisation/valgisation osteotomy and rotational osteotomy.

#### Varisation/valgisation osteotomy

The first osteotomy line is placed at the base of the femoral neck. To ensure stability, it is preferable to make this cut as horizontal as possible.

##### Femoral varisation

**Figure 4:**
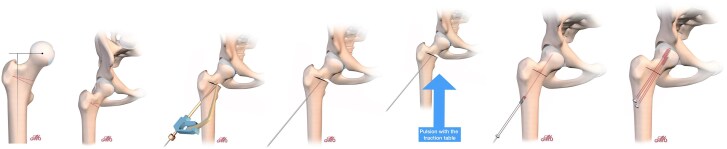
Varisation osteotomy.

For femoral varisation, a second osteotomy line is made above the first, creating a wedge of bone with an internal base corresponding to the desired correction angle. The wedge should not extend completely to the lateral part of the femur at the level of the greater trochanter, as the bone in this area will naturally crack upward, allowing for controlled correction. The oscillating saw should be halted at the insertion of the vastus lateralis, just below the trochanteric tubercle. A gentle application of force on the orthopaedic table will facilitate the natural closure of the osteotomy. If resistance is encountered, the lateral portion of the osteotomy can be completed with a chisel to encourage a controlled fracture at the greater trochanter (Fig. 4).

##### Femoral valgisation

**Figure 5:**
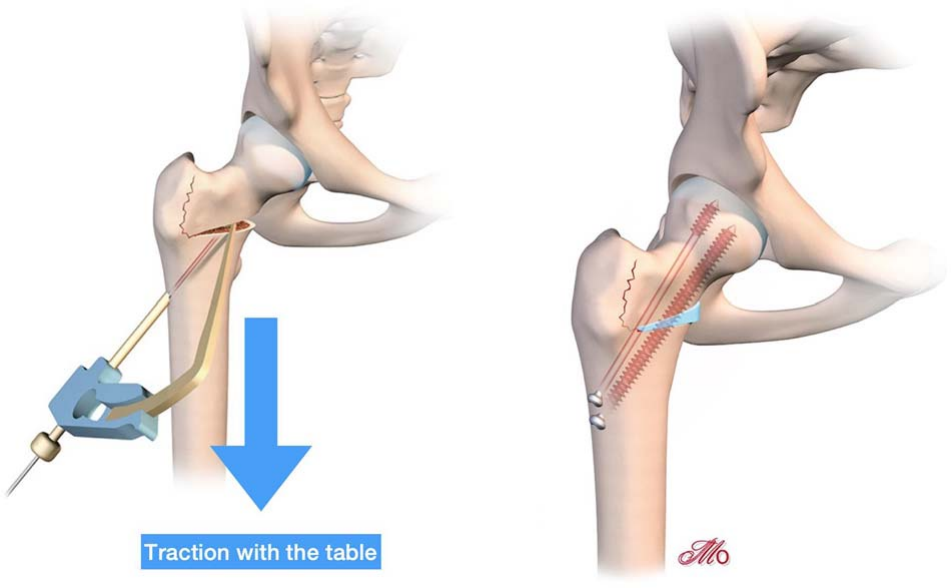
Valgisation osteotomy.

For valgisation osteotomy, a single osteotomy line is made on the inner half of the femur. Applying traction along the femoral axis naturally opens the osteotomy line. The cut should remain incomplete, with the lateral portion acting as a controlled fracture site. The medial portion is then opened further using traction on the orthopaedic table (Fig. 5).

#### Rotation/derotation osteotomy

Since rotation requires complete mobilisation of the upper femoral segment, a second osteotomy line is necessary to prevent mobilisation of the greater trochanter.

**Figure 6:**
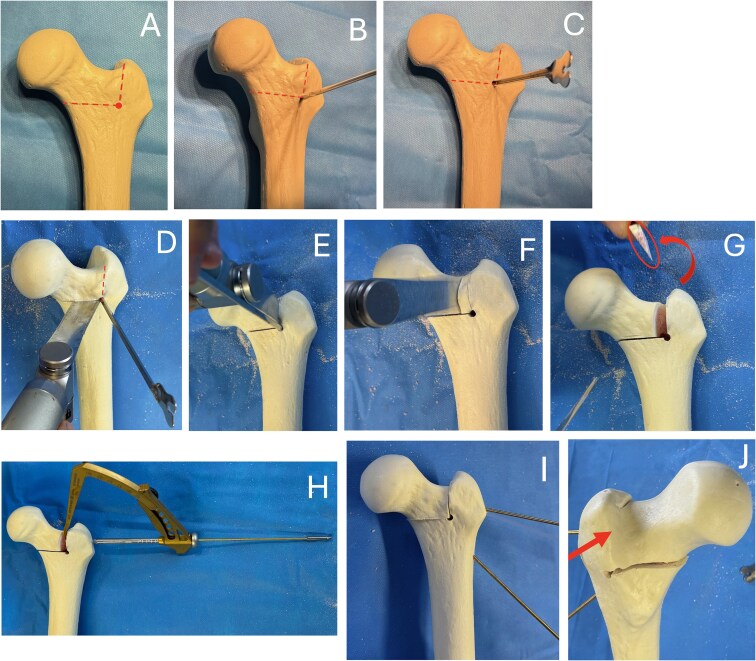
Femoral rotation osteotomy on sawbone model.

Before performing the osteotomy, a 4.5 mm drill bit is used to perforate the anterior cortex at the junction of the two planned osteotomy lines (Fig. 6B). This step, guided by the image intensifier, helps ensure accurate positioning of the cuts. In place of the drill bit, a grooved probe is introduced (Fig. 6C). Directed towards the horizontal osteotomy line, it provides protection for the hinge and assists in maintaining the correct alignment of the saw blade (Fig. 6D). The first osteotomy is a horizontal cut, starting above the lesser trochanter and extending to the lower insertion of the greater trochanter beneath the trochanteric tubercle. The second osteotomy is a vertical cut, perpendicular to the first, located just posterior to the femoral neck origin and terminating within the trochanter while preserving the vascular retinaculum. To avoid damaging the vascular structures when performing this second vertical osteotomy, it is advisable to cut only the anterior cortex. This preserves a posterior bony hinge that protects the medial circumflex vessels (Fig. 6J). The foot is then placed in internal rotation, and an osteotome is introduced into the vertical osteotomy line. The foot and knee are subsequently returned to a neutral position, while the osteotome maintains the femoral neck in internal rotation, opening the osteotomy. The posterior cortex naturally fractures, functioning as a controlled posterior hinge.

##### Femoral derotation

**Figure 7:**
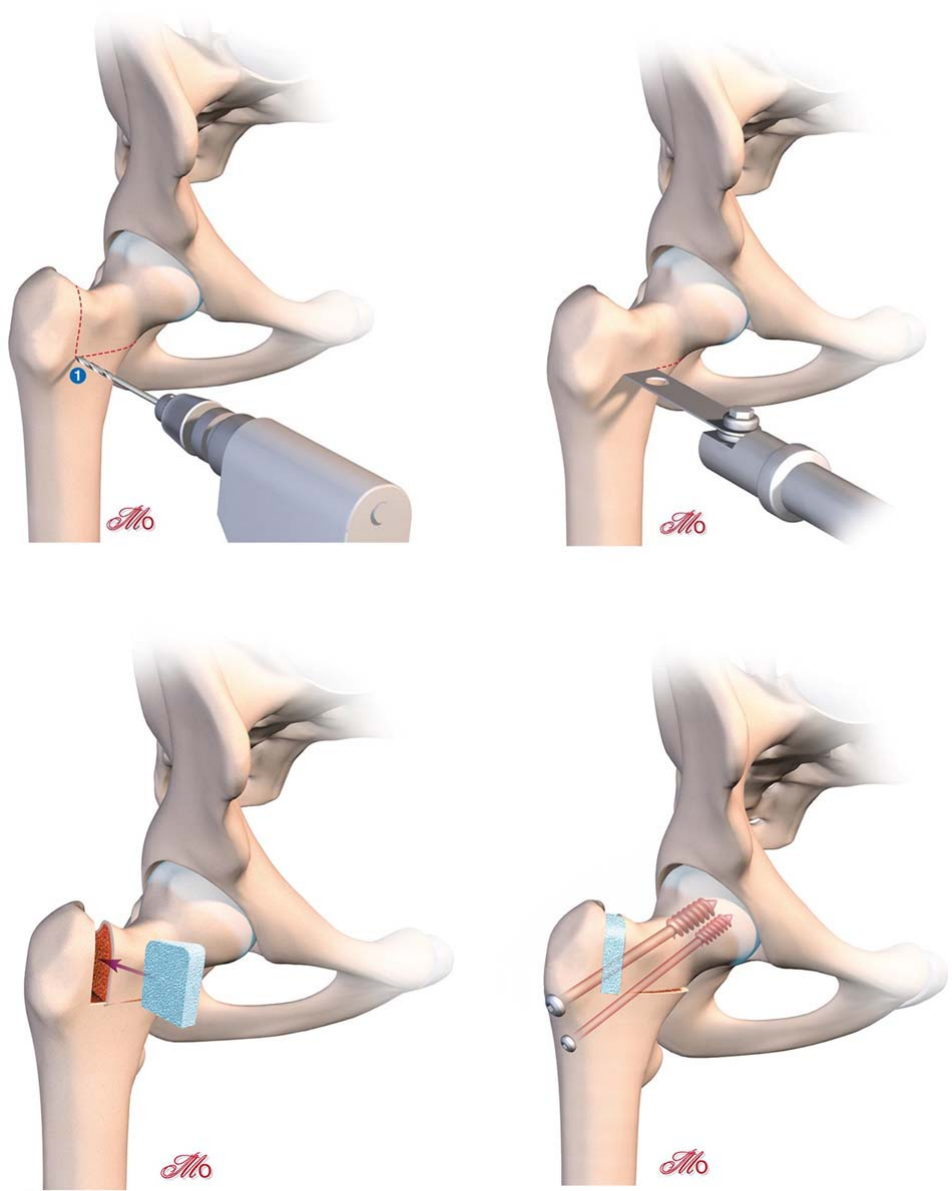
Derotation osteotomy: correction of hyper-anteversion.

The open osteotomy gap is systematically filled with a small allograft wedge to ensure structural integrity (Fig. 7).

##### Femoral rotation

Once the vertical osteotomy is completed, a second vertical cut is made, progressively more medial depending on the required degree of correction. This cut is directed towards the first osteotomy line, meeting it at the posterior cortex to create a wedge. The foot is then placed in maximal internal rotation, allowing the osteotomy to close naturally (Fig. 6 and Supplementary Fig. 1).

### Femoral fixation

**Figure 8:**
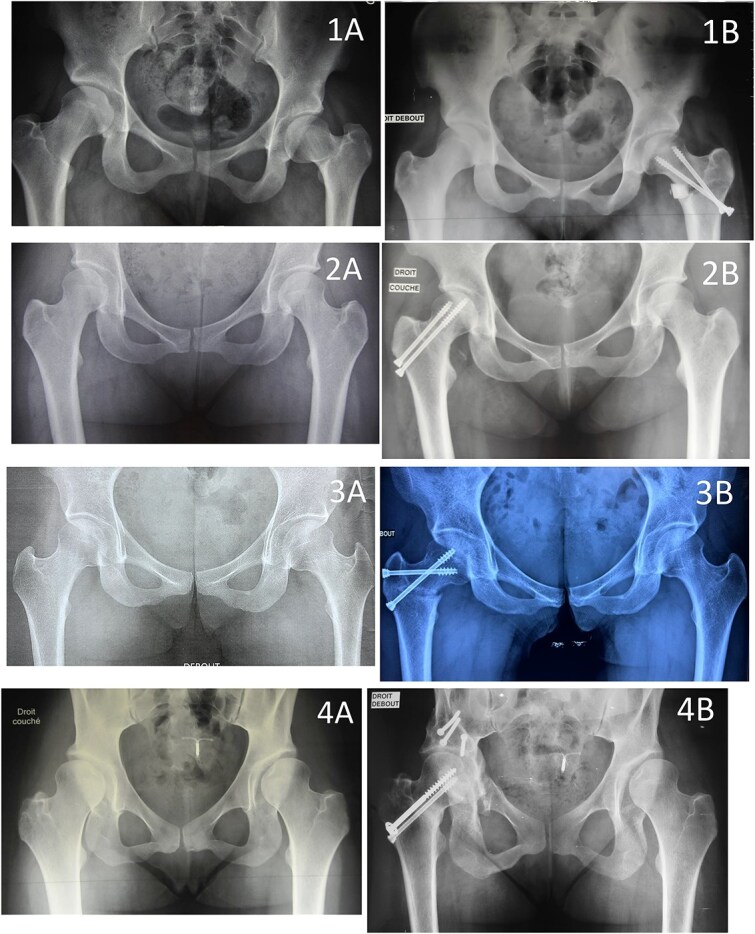
Examples of femoral neck osteotomies: (1) valgisation, (2) varisation, (3) anteversion, and (4) varisation combined with periacetabular osteotomy. (A) Preoperative. (B) Postoperative.

In closing osteotomies (femoral rotation or varisation), a tibial guide from cruciate ligament reconstruction is used to precisely guide a pin through the osteotomy line before applying compression with the orthopaedic table. Once compression is applied to fully close the osteotomy, the pin is advanced into the femoral head. With the pin already positioned, an 8 mm cannulated screw is inserted to stabilise the construct. A second screw is placed parallel to the first (Figs 4, 6, and 8).

In opening osteotomies (femoral derotation or valgisation), a hydroxyapatite wedge is inserted into the osteotomy gap to enhance stability. The screws are placed immediately after positioning the wedge (Figs 5, 7, and  8). For valgisation osteotomies, a fully threaded screw should be inserted as close as possible to the internal cortical bone and into the femoral neck, where bone quality is optimal. This ensures that the osteotomy gap does not collapse under load, preserving the graft. A second screw is positioned laterally and aligned parallel to the first. (Figs 5 and 8).

### Post-operative protocol

The post-operative protocol includes toe-touch weight-bearing for a duration of 45 days. No physiotherapy is required.

## DISCUSSION

Traditional femoral osteotomies have been performed *via* a lateral approach with plate fixation, necessitating vastus lateralis detachment, or through a superior approach for derotation osteotomies using an intramedullary nail, which requires passage through the gluteal muscles [4]. In contrast, our technique utilises an intermuscular plane, preserving muscle integrity. Another limitation of conventional approaches is the requirement for multiple incisions when combined with additional procedures such as periacetabular osteotomy, hip arthroscopy, or mosaicplasty [4, 11]. Our technique, in contrast, relies on a single small incision, allowing multiple procedures. If a periacetabular osteotomy is needed, the same incision can simply be extended to the iliac crest. In the late 1990s, Laude *et al.* introduced this approach for FAI treatment, highlighting its excellent visualisation of the anterior metaphysis and femoral epiphysis [12].

A significant advantage of our technique is its ability to accommodate both varisation/valgisation and rotation corrections simultaneously, an option not available with intramedullary fixation methods.

Additionally, the osteotomy line in our approach is positioned higher than in traditional techniques, preserving the natural morphology of the femoral metaphysis and diaphysis. This anatomical preservation is crucial if a hip prosthesis is required in the future [7].

However, this technique demands a high level of precision. In varisation and valgisation osteotomies, it is essential that the osteotomy line terminates in the greater trochanter. If the controlled fracture extends too proximally into the femoral neck, there is a risk of femoral neck mobilisation. Another important consideration is the proximity of the osteotomy to the circumflex femoral vessels. Dewar *et al.* reported that 82% of the femoral head’s blood supply comes from the medial circumflex artery, with 67% reaching it through the femoral neck [13]. Consequently, ligation of the lateral circumflex artery is not a significant concern. On the other hand, it is essential not to damage the medial circumferential package, which arrives from the posterior part of the neck first to the posterior surface of the tendon of the external obturator [14, 15]. During its intra-capsular passage, these vessels become clearly visible and must be identified before proceeding with the osteotomy. In varisation and valgisation osteotomies, the risk of vascular injury is minimal, as the vessels remain sufficiently distant from the horizontal osteotomy and are protected by the external obturator. In contrast, rotation osteotomies require careful placement of the vertical osteotomy line, which should remain lateral, close to the greater trochanter, without breaching the posterior cortex. Since the rotational hinge is located at this level, the posterior cortex will fracture naturally, making it unnecessary to cut through it. When these precautions are observed, the risk of femoral head necrosis appears negligible. In our experience, we have not encountered any cases of vascular compromise in our patients.

## Supplementary Material

Supplemental_Figure_1_hnaf041

## Data Availability

No new data were generated or analysed in present study.
